# Dynamic patterns of DNA methylation in the normal prostate epithelial differentiation program are targets of aberrant methylation in prostate cancer

**DOI:** 10.1038/s41598-021-91037-1

**Published:** 2021-06-01

**Authors:** Mark D. Long, Vineet K. Dhiman, Hayley C. Affronti, Qiang Hu, Song Liu, Dominic J. Smiraglia

**Affiliations:** 1 Department of Cancer Genetics and Genomics, Roswell Park Comprehensive Cancer Center, Buffalo, NY 14263 USA; 2 Department of Biostatistics and Bioinformatics, Roswell Park Comprehensive Cancer Center, Buffalo, NY 14263 USA

**Keywords:** Cancer genomics, Prostate cancer

## Abstract

Understanding the epigenetic control of normal differentiation programs might yield principal information about critical regulatory states that are disturbed in cancer. We utilized the established non-malignant HPr1-AR prostate epithelial cell model that upon androgen exposure commits to a luminal cell differentiation trajectory from that of a basal-like state. We profile the dynamic transcriptome associated with this transition at multiple time points (0 h, 1 h, 24 h, 96 h), and confirm that expression patterns are strongly indicative of a progressive basal to luminal cell differentiation program based on human expression signatures. Furthermore, we establish dynamic patterns of DNA methylation associated with this program by use of whole genome bisulfite sequencing (WGBS). Expression patterns associated with androgen induced luminal cell differentiation were found to have significantly elevated DNA methylation dynamics. Shifts in methylation profiles were strongly associated with Polycomb repressed regions and to promoters associated with bivalency, and strongly enriched for binding motifs of AR and MYC. Importantly, we found that dynamic DNA methylation patterns observed in the normal luminal cell differentiation program were significant targets of aberrant methylation in prostate cancer. These findings suggest that the normal dynamics of DNA methylation in luminal differentiation contribute to the aberrant methylation patterns in prostate cancer.

## Introduction

Current understanding of the prostatic tissue architecture suggests that the epithelial component of the prostate gland is stratified into several distinct cellular subtypes^1^. The non-secretory basal cells are found in the basal membrane of the glandular unit. These cells are considered androgen independent, and furthermore are thought to serve as stem or progenitor populations for other glandular cell components. Conversely, the secretory luminal cell population sits above the basal layer, producing and secreting the components of the prostatic fluid. These cells are androgen dependent, requiring stimulation of the androgen receptor (AR) by dihydrotestosterone (DHT) or other androgens for their function and survival. Thus, androgen signaling through the AR is a critical mediator of cellular differentiation and proliferative programs both during prostatic development and in the adult tissue.

AR dysfunction is central to prostate cancer (PCa) development and progression and hence the AR serves as a major non-surgical therapeutic target in the disease. While both basal and luminal cells have been implicated as possible cell-of-origin for PCa^2,3^, increasing evidence suggests that malignant transformation of the prostate most often arises from the luminal cell compartment. However, while its origins are founded within the context of the luminal cell, aggressive PCa is associated with a basal like transcriptome^4^, suggesting a reversion of the normal differentiation program.

The immortalized HPr1-AR non-malignant prostate epithelial cell line serves as a well-established model of normal androgen signaling^5–7^. We and others have confirmed that HPr1-AR cells express markers of basal and/or progenitor like cells such as KRT5, MYC and BCL2 in the absence of androgens. Exposure to androgens leads to suppression of such gene programs, dampens cellular growth, and instead promotes expression of luminal cell markers such as KRT8 and KRT18^6,8^. While expression patterns associated with this transition have been documented, to what extent epigenetic dynamics, such as shifts DNA methylation patterns, are associated with this process is unclear. We have previously utilized this model to develop a candidate understanding of androgen induced dynamic DNA methylation in normal prostate^7^. The goal of the current study was to elaborate on these findings by simultaneously mapping the dynamic transcriptome and DNA methylome associated with androgen induced luminal cell differentiation in HPr1-AR cells. Furthermore, we sought to assess to what extent dynamic methylation changes associated with the normal differentiation program are selected for aberrant methylation in PCa.

## Methods

### Cell culture

All cells were maintained at 37 °C and 5.0% CO_2_ using a cell culture incubator with UV contamination control (Sanyo). HPr1-AR cells were maintained in Keratinocyte-Serum Free Medium (KSF) containing human recombinant Epidermal Growth Factor 1–53 (EGF 1–53) and Bovine Pituitary Extract (BPE). Media was supplemented with 100 U/mL Penicillin–Streptomycin. DHT (sigma) was diluted in EtOH to 1000 × stocks in prior to treatments. Cells were treated with either EtOH or DHT (10 nM) for 1, 24 and 96 h.

### RT-qPCR

Quantitative real-time reverse transcription–polymerase chain reaction (RT-qPCR) was employed for detection of target mRNA transcripts as previously described^8^. All targets were detected using either pre-designed TaqMan Gene Expression Assays (Thermo Fisher Scientific; *AR*, *GAPDH*) or pre-designed KiCqStart^®^ qPCR primer sets (Sigma-Aldrich; *DPP4*, *S100P*, *KRT8*, *KRT18*, *STEAP4*, *TMEM56*, *KRT5*, *CPA4*) using a final primer concentration of 500 nM. Primer sequences are provided in Supplementary Table S1.

### Immunoblotting

Total cellular protein was isolated from exponentially growing cells for determination of target protein expression as previously described^8^.

### RNA-sequencing

To determine global changes in mRNA, HPr1-AR cells exposed to DHT (10 nM) for 1, 24 and 96 h were analyzed by RNA-seq (n = 4 per condition). Raw sequence reads (75 bp paired end, > 45 × 10^6^ average reads/sample) were aligned to the human genome (hg19) using tophat2. Aligned reads were used to determine expression counts via featurecounts, followed by a standard DESeq2^9^ pipeline for data normalization and differential expression analysis.

### Whole genome bisulfite sequencing

To determine global changes in DNA methylation, HPr1-AR cells exposed to DHT (10 nM) for 1, 24 and 96 h were analyzed by whole genome bisulfite sequencing (WGBS). Raw sequencing reads were aligned to the human genome (hg19) and methylation levels called using bismark^10^. Only CpG sites with a minimum coverage of 20 × were considered. Dynamic methylation positions were identified using the following criteria; methylation changes over time were progressive (0 h < 1 h < 24 h < 96 h, or 0 h > 1 h > 24 h > 96 h) with a minimum overall change of at least 20% (abs(96–0 h) > 20%).

### Methylation capture sequencing

To confirm methylation levels measured by WGBS, replicate samples were analyzed by TruSeq Methyl Capture sequencing (methyl-capture seq). Methylation levels were identified in the same manner as that utilized for WGBS. Of the 11,191,175 sites profiled at 20 × coverage by WGBS, 775,019 were also covered by MethylCapture-seq at 10 × coverage. Briefly, the TruSeq Methyl Capture EPIC Library Prep Kit from Illumina was used to prepare the sequencing library from 500 ng of genomic DNA. The DNA was sheared and then ends repaired prior to adenylation of 3′ ends and ligation of adapters. The fragments were then hybridized to the capture probes. Captured DNA was then bisulfite treated prior to amplification of the library and sequencing.

### Determining methylation variability at TSS regions

For all TSS loci (RefSeq), TSS regions were defined as ± 5 kb. The methylation dispersion (standard deviation) of each CpG position was calculated across samples, and the mean dispersion for all CpG positions found within each TSS region was calculated. For further analyses, only TSS regions with at least 20 CpG positions were considered. For GSEA analysis, only TSS regions of genes that were expressed at detectable levels in HPr1-AR cells were used.

### Gene set enrichment analysis (GSEA)

GSEA was performed using the fgsea package in R. Pre-ranked gene lists were produced using DESeq2 determined differentially expressed genes (using Wald statistic as ranking parameter), or from TSS region methylation variability (using mean variation as ranking parameter) for expression and DNA methylation data, respectively. Visualization of enriched gene sets was done using EnrichmentMap in Cytoscape v3.7.1. To maintain an unbiased assessment of functional enrichment, a set of 5407 gene sets were queried from the Molecular Signatures Database (MSigDB), including all HALLMARK, canonical pathways (including KEGG, REACTOME and BIOCARTA gene sets) and GO terms (biological processes). Also included were gene sets corresponding to defined human luminal and basal prostate epithelial cell markers^4^ and HPr1-AR cells treated with androgen for 24 h^6^.

### Motif enrichment analysis

Motif assessment was performed on dynamic and non-dynamic DNA methylation positions (± 20 bp) using the findMotifsGenome.pl tool available from the HOMER (Hypergeometric Optimization of Motif EnRichment) suite.

### Publically available data

All analyses were undertaken using the R platform for statistical computing^11^ (version 3.6.0) and a range of library packages were implemented in Bioconductor^12^. Publically available data (ChromHMM regions, ChIP-seq data, WGBS from human samples, 450 k array data from TCGA-PRAD cohort) were downloaded from the Gene Expression Omnibus (GSE76336, GSE73994, GSE104789) or from FireBrowse (http://firebrowse.org/). Gene (RefSeq) and CpG island (UCSC) annotations were obtained directly from the UCSC Table Browser. TSS regions were determined as each TSS location ± 5 kb. CpG island regions were determined as the boundaries of each island ± 500 bp.

## Results

### Androgen induces transcriptional patterns associated with luminal differentiation

**Figure 1:**
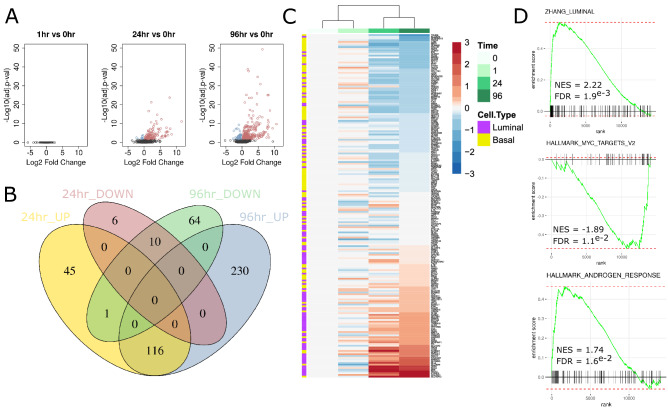
Global transcriptional patterns associated with androgen induced differentiation in HPr1-AR cells. (**A**) Volcano plots depicting expression changes at each time point (1 h, 24 h, 96 h) relative to untreated cells (n = 4 at each time point). (**B**) Venn diagram of DEGs identified at 24 and 96 h of DHT exposures. (**C**) Heatmap of top 100 luminal and basal differentiating genes as identified by Zhang et al.^4^. (**D**) Select enrichment plots from GSEA analysis comparing 96 h of DHT exposure to 0 h.

In validation of the model system, DHT exposure (10 nM) resulted in suppression of cellular growth, predictable shifts in cytokeratin profiles (increase in CK8, decrease in CK5) and gene expression changes that are indicative of previously reported androgen responses in HPr1-AR cells (Supplementary Fig. S1A–C). To understand dynamic changes in the transcriptome along the time course of androgen induced differentiation of HPr1-AR cells, RNA-seq was applied at 0, 1, 24 and 96 h of exposure to DHT. Expression changes (FDR < 0.05, FC > 1.5) were substantial at 24 h (178 differentially expressed genes (DEGs); 162 upregulated, 16 downregulated), but broader at 96 h (421 DEGs; 346 upregulated, 75 downregulated) (Fig. 1A,B). No DEGs were observed at 1 h of exposure. A majority (70.7%) of DEGs detected at 24 h of DHT exposure were maintained at 96 h suggesting a progressive and stable shift in global transcription. Gene expression changes largely confirmed those observed in a previous study of androgen response in HPr1-AR cells measured by microarray^6^ (Supplementary Fig. S1D). Notably, differential expression significantly aligned with gene sets that define human basal and luminal epithelial cells in the human prostate^4^, with the most upregulated genes strongly enriched for expression patterns specific to the luminal cells of human prostate (Fig. 1C,D). Unbiased functional annotations of expression patterns to 5407 gene sets available from the Molecular Signatures Database confirmed this, as luminal cell differentiating genes were amongst the top 3 overall hits. Other notable functional enrichments were observed for hypoxia and steroid response pathways, as well as a suppression of MYC and NFKB signaling (Fig. 1D, Supplementary Fig. S1E,F, Supplementary Table S2).

### DNA methylation dynamics associate with androgen induced luminal differentiation genes

To examine the changes in DNA methylation patterns associated with the androgen induced differentiation program in HPr1-AR cells, whole genome bisulfite sequencing was applied. In total, 11,191,175 CpG sites were profiled over time (minimum 20 × coverage at each time point). Hierarchical clustering and PCA analyses revealed decreasing similarity of global androgen induced methylation patterns relative to untreated cells over time, suggesting broad methylation dynamics associated with differentiation (Supplementary Fig. S2A). To confirm methylation calls as determined by WGBS, replicate samples were profiled by Targeted methylation sequencing (Methyl-Capture Seq). Using a 10 × coverage threshold, 775,019 CpG sites profiled by WGBS were obtained. Methylation levels as determined by Methyl-Capture Seq were highly correlated to those determined by WGBS, increasing with optimal coverage (r > 0.95) (Supplementary Fig. S2B).

**Figure 2:**
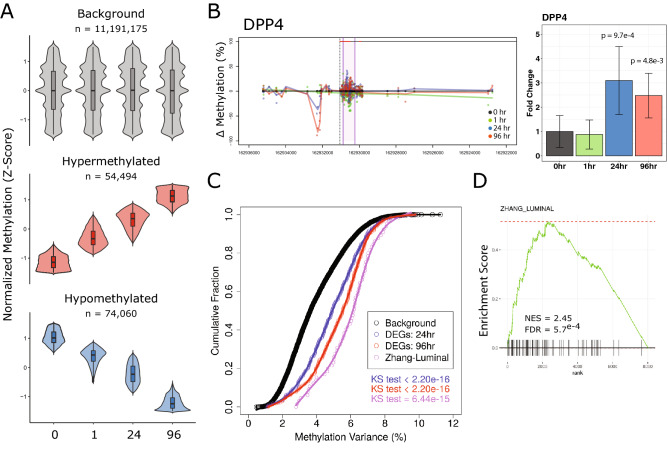
Dynamic DNA methylation associated with androgen induced differentiation. (**A**) Normalized (Z-score) plots of background CpG sites and identified dynamic methylation positions. (**B**) Candidate example of the luminal associated *DPP4* gene found to be associated with variable methylation and altered gene expression. Left: Genome view of DNA methylation changes relative to 0 h. Each dot represents an individual CpG at a given time point, indicated by color. Right: *DPP4* expression levels represented as fold change relative to 0 h. (**C**) Cumulative distribution plots showing the distribution of variance observed of methylation over time of all CpG sites compared to that observed for DEGs (24 h and 96 h) as well as for luminal associated genes. Differences in distributions were tested by Kolmogorov–Smirnov test. (**D**) Gene set enrichment was performed using androgen induced TSS methylation variance as a ranking parameter. Enrichment plot of luminal associated genes from GSEA of dynamic methylation of TSS regions.

To reveal dynamic sites of methylation, the following criteria were applied; methylation changes over time were progressive (e.g. 0 h < 1 h < 24 h < 96 h) with a minimum overall change of at least 20%. By these thresholds, 128,554 dynamic methylation positions (DMPs) were identified. A slight majority of DMPs were associated with loss of methylation (54,494 hypermethylated; 74,060 hypomethylated) (Fig. 2A). To examine how gene methylation patterns associated with gene expression, we determined the total time dependent variability of methylation at transcriptional start site (TSS) regions (± 5 kb) genome wide. A number of the most variable regions were observed proximal to luminal cell associated genes. For instance, 19 of the 96 luminal cell associated genes had methylation variance exceeding the 90th percentile of TSS methylation variance observed genome wide (*SLC26A4*, *PDE8B*, *ASRGL1*, *FGF13*, *SYTL2*, *PPP1R9A*, *GLYATL1*, *SNAP91*, *COL25A1*, *BMPR1B*, *SERHL2*, *KIAA1324*, *ARHGAP6*, *DPP4*, *GFPT2*, *GPR98*, *ATP13A4*, *SLC35F3*, and *RTN1*). The dynamic methylation pattern at the TSS of *DPP4* is shown in Fig. 2B. Integration of global expression changes with methylation dynamics revealed significantly higher variability at genes that were differentially expressed upon DHT exposure relative to the background transcriptome (Fig. 2C). Notably, this phenomenon was even greater at genes specific to luminal cell populations in human prostate tissue. This was confirmed by unbiased GSEA using DNA methylation variation as a ranking parameter, as luminal cell specific genes were amongst the most enriched gene sets for DNA methylation dynamics (Fig. 2D, Supplementary Table S3, Supplementary Fig. S3).

### Dynamic methylation enriches for polycomb repressed regions and transcription factor binding

**Figure 3:**
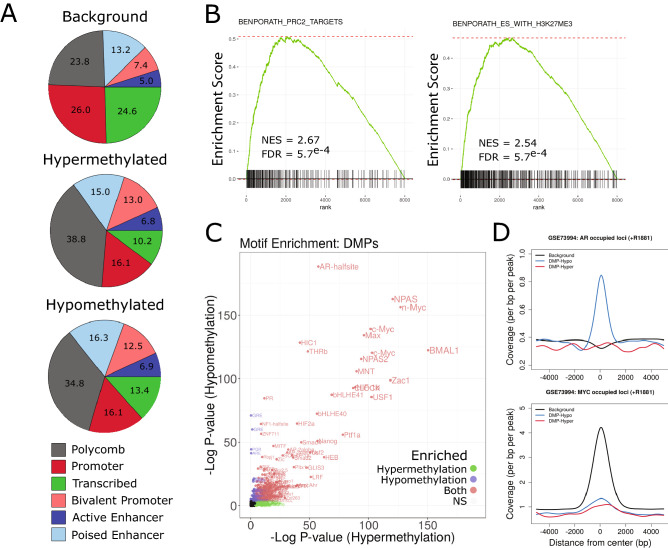
Dynamic methylation enriches for specific functional genomic loci and transcription factor binding. (**A**) Relative distributions of background and DMP sites to chromHMM identified regions observed in PrEC cells^13^. (**B**) Enrichment plots showing representative gene sets of Polycomb complex and H3K27me3 regulation pathways, two of the most frequently observed groupings from GSEA of dynamic methylation associated genes (Supplementary Fig. S3). (**C**) Motif enrichment was assessed at DMP positions (relative to background regions) using HOMER. (**D**) DNA binding profiles for AR and MYC in the presence of androgen were obtained (GSE73994) and enrichment at background CpG and DMP centered loci were assessed.

Next, we sought to characterize the functional genome associated with androgen induced methylation dynamics. Utilizing ChromHMM defined chromatin state profiles previously generated in non-malignant prostate epithelial cells (PrEC)^13^, we observed significant enrichment for annotated DMPs within Polycomb repressed regions, as well as in promoter regions associated with bivalency (Fig. 3A). Interestingly, this was true for both hypermethylated and hypomethylated sites as proportions between DMP groups were not substantially different. We detected strong enrichment for gene sets associated with Polycomb complex regulation (e.g. BENPORATH_PRC2_TARGETS) as well as it’s functional mark H3K27me3 (e.g. MIKKELSON_ES_WITH_H3K27ME3) in GSEA of TSS region methylation variance (Fig. 3B, Supplementary Fig. S3), further supporting the observed genomic distributions of DMPs in Polycomb repressed regions. Additionally, simultaneous enrichments for genes associated with H3K4me3 (e.g. MIKKELSON_ES_LCP_WITH_H3K4ME3) are supportive of DMPs association with regions of bivalency.

Motif analysis revealed significant enrichment of AR, MYC and hypoxia inducible factor (HIF) binding elements at DMPs relative to all CpG positions (Fig. 3C), reflective of GSEA of transcriptional profiles (Fig. 1D). Type I nuclear receptor (AR, GR, PGR) motif elements were enriched to a greater degree at hypomethylated than at hypermethylated DMP sites. Strong enrichment for enhancer-box (E-box) motif containing factors (MYC, NPAS, BMAL1) was observed to a similar degree at DMPs of both gain and loss. To address how dynamic methylation might affect genomic occupancy of these factors in prostate cells, ChIP-seq data for AR and MYC in the presence of androgen^14^ were queried. Indeed, AR binding was specifically enriched for sites of hypomethylation dynamics, suggesting a direct relationship between androgen directed occupancy and DNA methylation dynamics (Fig. 3D). Conversely, MYC binding was not enriched at DMPs, despite the strong association of total CpG positions with MYC binding due to the presence of a CpG dinucleotide within the E-box motif. In other words, regions of androgen induced methylation dynamics specifically exclude MYC occupancy. Thus, androgen induced DNA methylation dynamics seem to play an important and concerted role in the genomic distributions of both AR and MYC in prostate cell differentiation that is consistent with promoting AR driven gene regulation and antagonizing MYC driven gene regulation.

### Sites of normal luminal associated methylation dynamics are disrupted in prostate cancer

**Figure 4:**
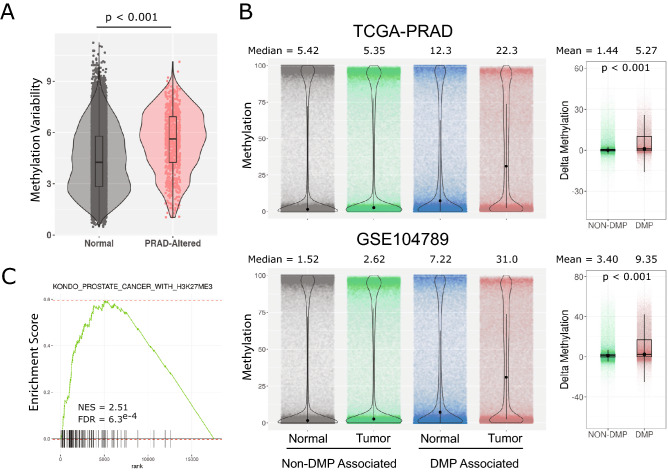
Regions of dynamic methylation associate with cancer susceptibility loci. (**A**) Overall androgen induced methylation variation over time was assessed for all TSS of genes expressed in HPr1-AR cells. The methylation variance of a set of genes previously reported to be associated with altered methylation in prostate cancer^15^ was examined and compared to background genes. Differences in distributions were determined by Wilcox test. (**B**) CpG island regions (UCSC) were compiled and parsed into DMP or Non-DMP associated regions. The overall methylation distributions of each group of regions was examined across normal and tumor samples from both the TCGA-PRAD and GSE104789 cohorts. (**C**) Select enrichment plot showing high association of methylation variable genes in HPr1-AR cells with genes associated with altered H3K27me3 in PCa.

A common characteristic of cancer development is the hypermethylation of CpG islands, often in gene promoters. DNA methylation patterns critical to androgen mediated differentiation in prostate epithelia may be prone to disruption in malignant transformation. To examine this question, methylation patterns from human prostate cancer cohorts were queried. In the first instance, methylation data from The Cancer Genome Atlas prostate adenocarcinoma (TCGA-PRAD) cohort were surveyed. Analysis of TSS regions (± 5 kb) of a previously identified set of genes shown to be associated with cancer specific aberrant promoter methylation in a meta-analysis of prostate cancer studies^15^ revealed a significantly higher degree of androgen induced methylation variance than that observed across the genome (Fig. 4A). Thus, promoters that become aberrantly methylated in prostate cancer have more androgen induced methylation dynamics.

We next asked if DMP associated CpG islands, regardless of genomic position, exhibited greater tumor associated hypermethylation than non-DMP associated islands. We examined CpG island methylation in both the TCGA-PRAD cohort, as well as a recent study that applied WGBS to 11 prostate cancers and 4 matching normal tissues^16^, after categorizing each CpG island as DMP associated or non-DMP associated. We found that non-DMP associated CpG islands had a minimal shift of overall methylation levels in tumors relative to normal tissue (Fig. 4B). However, DMP associated CpG islands displayed marked tumor associated hypermethylation in both cohorts, suggesting that islands marked by androgen regulated methylation dynamics are hyper susceptible to methylation in cancer. In addition, GSEA of methylation dynamic TSS loci in HPr1-AR cells strongly enriched for genes associated with cancer, including prostate cancer (e.g. WALLACE_PROSTATE_CANCER_RACE_UP) and specifically PCa with elevated H3K27me3 (KONDO_PROSTATE_CANCER_WITH_H3K27ME3) (Fig. 4C, Supplementary Fig. S3). In total, these studies indicate that genomic regions with androgen driven methylation dynamics in a model of luminal cell differentiation are preferred targets of aberrant methylation in prostate cancer.

## Discussion

Several recent studies have examined the cellular constitution of the human prostate in detail^1,17^. However to date, the DNA methylation dynamics and their relationships to transcriptional programs associated with luminal cell differentiation in the prostate remain enigmatic. To address this gap in knowledge, we applied an integrative approach, combining RNA-seq and WGBS at multiple time points along the androgen induced differentiation of the HPr1-AR cell model.

Using unbiased functional annotation approaches we confirm that global androgen induced expression patterns significantly associate with known luminal cell differentiation genes identified in human prostate tissues. Furthermore, we identify that this differentiation program is associated with elevated hypoxic and steroid responses, as well as suppression of MYC and IFN signaling. Repression of MYC expression and signaling is a critical component of the normal androgen induced differentiation program in prostate epithelial cells^18^.

Identification of variable DNA methylation associated with androgen induced differentiation using progressive criteria revealed 128,554 dynamic methylation positions. Importantly, we observed a significant degree of DNA methylation dynamics at regions proximal to differentially expressed genes, showing for the first time a global association between methylation dynamics and the androgen mediated differentiation program in prostate epithelia. Furthermore, we establish that genes previously identified as specific to human prostatic luminal populations have a high degree of androgen dependent DNA methylation dynamics, suggesting that precise regulation of DNA methylation at critical loci are required for the maintenance of proper cellular identity in the prostate.

Methylation dynamics were enriched at Polycomb repressed regions as well as at promoters marked with bivalency, suggesting targeted epigenomic remodeling of specific genomic domains. Polycomb complexes have been shown to specifically recruit DNMT enzymes through EZH2 in order to modulate DNA methylation levels at proximal loci^19^, and furthermore EZH2 plays a major role in the maintenance of progenitor populations and luminal cell determination in mammary glands^20^. Promoters associated with bivalency, or the presence of both activating and repressive histone marks, are commonly found near genes involved in developmental programs and are associated with dynamic DNA methylation patterns induced by differentiation^21^.

DMPs were strongly enriched for binding motifs of AR and MYC. We previously showed that androgen exposure results in DNA methylation dynamics at candidate AR regulated loci, coincident with recruitment of methylation modifying enzymes such as TET1, modulation of protein complex occupancy and transcriptional output^7^. We now confirm DNA dynamics at AR binding sites as a general phenomenon of luminal cell differentiation. A recognized observation of transcriptional patterns associated with this process is the antagonistic behavior of the AR and MYC expression and signaling^8,14^. It is therefore notable that DNA methylation dynamics are also observed to a large extent at E-box elements bound by MYC/MAX and other basic helix-loop-helix (bHLH) factors such as CLOCK, BMAL1 and NPAS1/2. Importantly, the E-box element contains a CpG dinucleotide, the methylation status of which modulates MYC occupancy^22,23^. This suggests that the DNA methylation dynamics driven by androgen exposure may play an important role in determining the binding capacity of AR and MYC at such genomic positions during differentiation. This idea was confirmed in the analysis of global binding patterns of these factors in prostate cells, where androgen induced AR binding was observed specifically at regions of hypomethylation dynamics, whereas MYC binding was excluded from dynamic loci. These findings are consistent with the finding that MYC binding is negatively affected by the presence of DNA methylation^23^, and implicates epigenetic exclusion of MYC as a major contributor to the anti-proliferative effect of androgens in normal prostate epithelia. Additional enrichments suggest shifted accessibility of other factors including HIFs, also in line with a strong induction of hypoxia and HIF1α signaling in the androgen mediated transcriptome^24^.

An important conclusion from the current study is that regions of androgen induced dynamic methylation associated with normal luminal differentiation are preferential targets of aberrant hypermethylation in PCa. This phenomenon was apparent both at TSS loci previously determined to display tumor associated methylation changes in PCa as well as at CpG island regions, where the presence of DMPs was associated with tumor hypermethylation. Global assessment of DNA methylation patterns in PCa samples is limited. However, we were able to confirm these findings in two independent cohorts, one a large cohort with limited coverage (TCGA-PRAD) and another with limited samples but denser coverage of the DNA methylome^16^. These observations further support the importance of androgen mediated methylation dynamics in the maintenance of normal luminal cell function in the prostate and suggest that the deterioration of dynamic regulation at these loci is associated with malignant transformation. The fact that genomic regions exhibiting dynamic methylation changes in the normal differentiation setting are preferred targets of aberrant methylation in prostate cancer raises the possibility that the normal dynamic regulation of methylation makes these regions hyper-susceptible to aberrant methylation.

## Supplementary Information

Supplementary Information.

## Data Availability

All data associated with the current study has been deposited in the gene expression omnibus under accession #, s GSE174618 for RNA-seq; GSE174662 for Methyl Capture; GSE174711 for WGBS.
