# Supplementary material for: Dynamic patterns of DNA methylation in the normal prostate epithelial differentiation program are targets of aberrant methylation in prostate cancer
Source: Sci Rep. 2021 Jun 1;11:11405. doi: 10.1038/s41598-021-91037-1 (PMC8169877; doi:10.1038/s41598-021-91037-1)
Supplement: Supplementary file 1 — Supplementary Information. [file 41598_2021_91037_MOESM1_ESM.pdf]

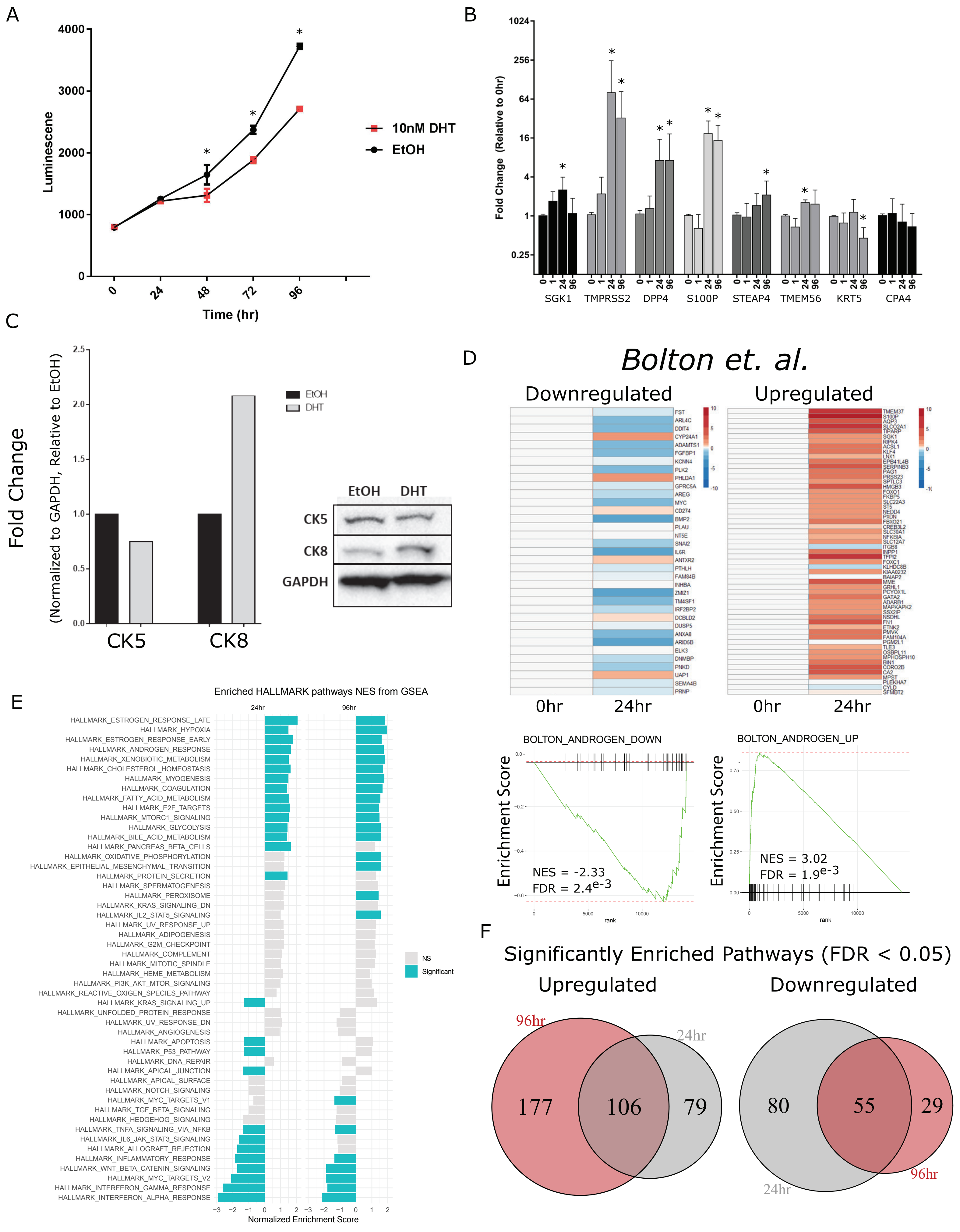

A
Luminescene
Time (hr)
10nM DHT
EtOH
B
Fold Change (Relative to 0hr)
SGK1
TMPRSS2
DPP4
S100P
STEAP4
TMEM56
KRT5
CPA4
C
Fold Change
(Normalized to GAPDH, Relative to EtOH)
EtOH
DHT
CK5
CK8
GAPDH
D
Bolton et. al.
Downregulated
Upregulated
0hr
24hr
BOLTON_ANDROGEN_DOWN
BOLTON_ANDROGEN_UP
Enrichment Score
NES = -2.33
FDR = 2.4e-3
NES = 3.02
FDR = 1.9e-3
rank
E
Enriched HALLMARK pathways NES from GSEA
24hr
96hr
HALLMARK_ESTROGEN_RESPONSE_LATE
HALLMARK_HYPOXIA
HALLMARK_ESTROGEN_RESPONSE_EARLY
HALLMARK_ANDROGEN_RESPONSE
HALLMARK_XENOBIOTIC_METABOLISM
HALLMARK_CHOLESTEROL_HOMEOSTASIS
HALLMARK_MYOGENESIS
HALLMARK_COAGULATION
HALLMARK_FATTY_ACID_METABOLISM
HALLMARK_E2F_TARGETS
HALLMARK_MTORC1_SIGNALING
HALLMARK_GLYCOLYSIS
HALLMARK_BILE_ACID_METABOLISM
HALLMARK_PANCREAS_BETA_CELLS
HALLMARK_OXIDATIVE_PHOSPHORYLATION
HALLMARK_EPITHELIAL_MESENCHYMAL_TRANSITION
HALLMARK_PROTEIN_SECRETION
HALLMARK_SPERMATOGENESIS
HALLMARK_PEROXISOME
HALLMARK_KRAS_SIGNALING_DN
HALLMARK_IL2_STAT5_SIGNALING
HALLMARK_UV_RESPONSE_UP
HALLMARK_ADIPOGENESIS
HALLMARK_G2M_CHECKPOINT
HALLMARK_COMPLEMENT
HALLMARK_MITOTIC_SPINDLE
HALLMARK_HEME_METABOLISM
HALLMARK_PI3K_AKT_MTOR_SIGNALING
HALLMARK_REACTIVE_OXIGEN_SPECIES_PATHWAY
HALLMARK_KRAS_SIGNALING_UP
HALLMARK_UNFOLDED_PROTEIN_RESPONSE
HALLMARK_UV_RESPONSE_DN
HALLMARK_ANGIOGENESIS
HALLMARK_APOPTOSIS
HALLMARK_P53_PATHWAY
HALLMARK_DNA_REPAIR
HALLMARK_APICAL_JUNCTION
HALLMARK_APICAL_SURFACE
HALLMARK_NOTCH_SIGNALING
HALLMARK_MYC_TARGETS_V1
HALLMARK_TGF_BETA_SIGNALING
HALLMARK_HEDGEHOG_SIGNALING
HALLMARK_TNFA_SIGNALING_VIA_NFKB
HALLMARK_IL6_JAK_STAT3_SIGNALING
HALLMARK_ALLOGRAFT_REJECTION
HALLMARK_INFLAMMATORY_RESPONSE
HALLMARK_WNT_BETA_CATENIN_SIGNALING
HALLMARK_MYC_TARGETS_V2
HALLMARK_INTERFERON_GAMMA_RESPONSE
HALLMARK_INTERFERON_ALPHA_RESPONSE
NS
Significant
Normalized Enrichment Score
F
Significantly Enriched Pathways (FDR < 0.05)
Upregulated
Downregulated
96hr
24hr
177
106
79
80
55
29

Supplementary Figure 1: Identifying global transcriptional and DNA methylation patterns in androgen driven differentiation. A) Proliferation of HPr1-AR cells treated with DHT (10nM) or vehicle control (EtOH) up to 96hr post-treatment. The data represent experiments performed in biological triplicates. **B)** RT-qPCR assessment of known DHT responsive genes in HPr1-AR cells. **C)** Cytokeratin profiling (immunoblot) of two well established markers of basal cell populations (CK5) and luminal cell populations (CK8). **D)** Heatmap and enrichment plots comparing observed expression changes by RNA-seq (24hr) to those previously reported in HPr1-AR cells by microarray profiling (24hr)[6]. **E)** Summary of all HALLMARK pathway enrichments determined by GSEA analysis (24hr and 96hr relative to 0hr). Normalized Enrichments Scores (NES) are shown on the x-axis. Significant enrichments ($p.adj < 0.1$) are shown in blue; non-significant enrichments are shown in grey. **F)** Venn diagram depicting number of significantly enriched pathways from complete GSEA analysis between 24hr and 96hr of DHT exposure.

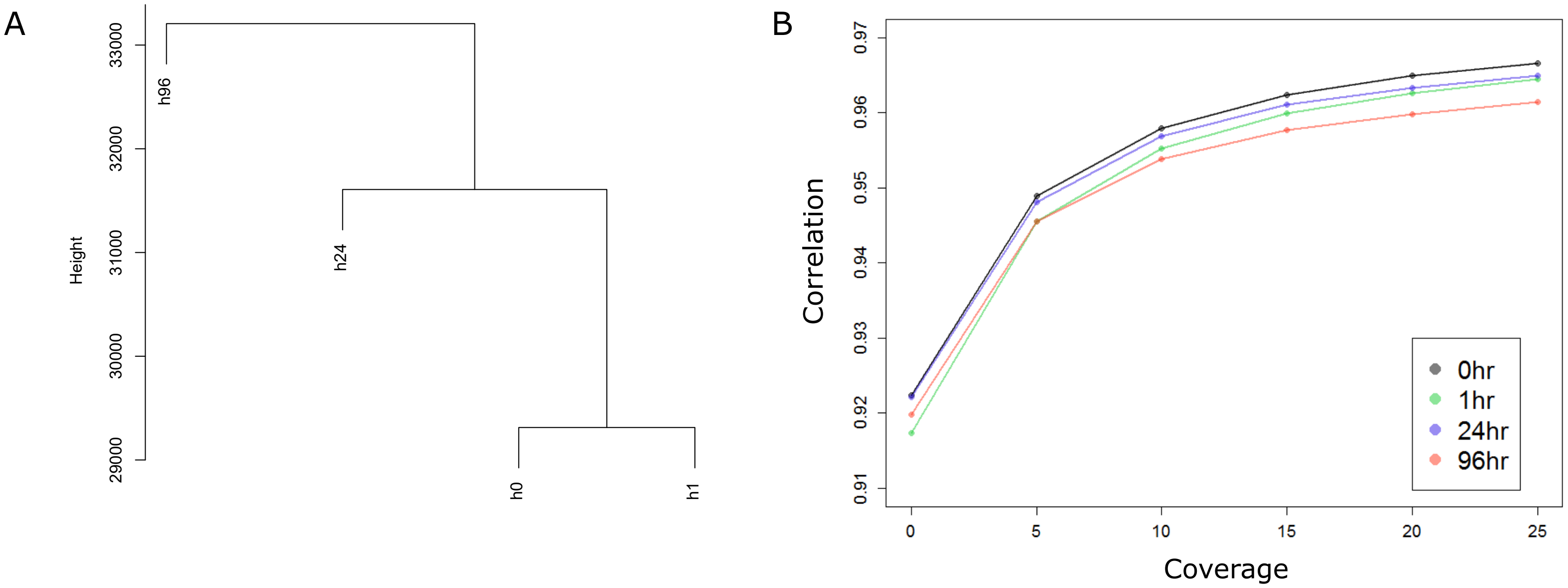

Supplementary Figure 2: Confirmation of whole-genome bisulfite sequencing approach using TruSeq Methyl Capture sequencing A) Hierarchical clustering of WGBS samples. Euclidean distance was used as a measure of similarity. **B)** Correlation of WGBS methylation calls to those identified in Methyl-Capture seq approach at different levels of sequencing coverage.

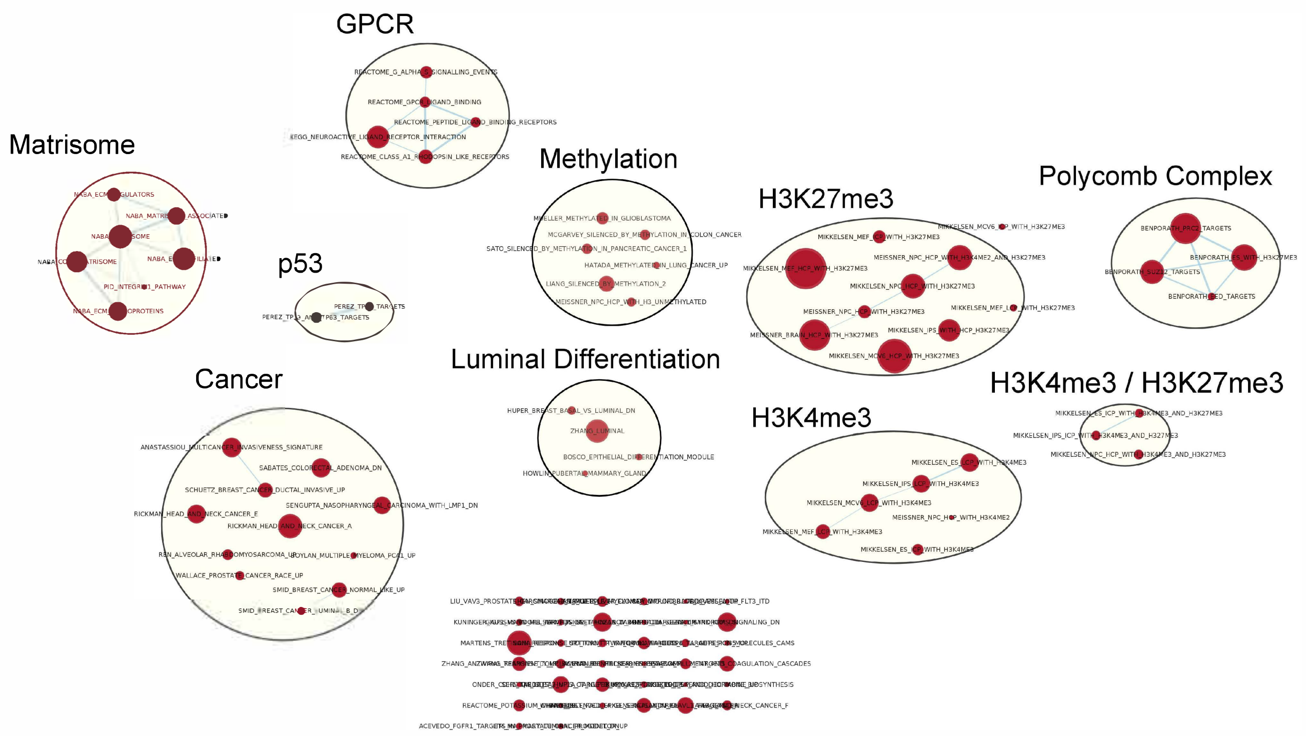

Supplementary Figure 3: Enrichment map of significantly enriched pathways associated with androgen induced DNA methylation dynamics. GSEA was performed, using overall variance at TSS loci genome wide, to reveal functional enrichment of genes associated with DNA methylation dynamics. Shown are significantly enriched gene sets (FDR < 0.05). Size of nodes represent normalized enrichment scores, and edges represent an overlap of at least 35% between two gene sets.

Table S1: Primers sequences used in study

Gene	Forward	Reverse	Amplicon Length
DPP4	GAAGAGAGGATTCCAAACAAC	CATTGTTCCAAACATATGCC	79
S100P	TCTGAATCTAGCACCATGAC	CATCCTTGTCTTTTCCACTC	172
KRT8	ACGAATTTGTCCTCATCAAG	CCGGATCTCCTCTTCATATAG	128
KRT18	GGAAGTAAAAGGCCTACAAG	GTACTTGTCTAGCTCCTCTC	154
STEAP4	TGATTCATATGTGGCTTTGG	CAGTTTGGACTGGACAAATC	124
TMEM56	ATGGAACTCAAGGGTAGTATC	TGGACAAATCAGAAATGAGG	170
KRT5	AGTTTGTGATGCTGAAGAAG	GTTAATCTCATCCATCAGTGC	92
CPA4	AGAAATGGAGACGAGATCAG	GGGAGATTTCCAGAAATTGAG	78

Table S2: Top 30 significantly enriched upregulated and downregulated pathways in HPr1-AR cells exposed to DHT for 96 hours.

pathway	pval	padj	NES
BOLTON_ANDROGEN_UP	1.40E-04	7.67E-03	3.02
MENSE_HYPOXIA_UP	1.36E-04	7.67E-03	2.33
ZHANG_LUMINAL	1.28E-04	7.67E-03	2.22
PYEON_CANCER_HEAD_AND_NECK_VS_CERVICAL_UP	1.24E-04	7.67E-03	2.20
ELVIDGE_HIF1A_TARGETS_DN	1.37E-04	7.67E-03	2.15
IIZUKA_LIVER_CANCER_PROGRESSION_G2_G3_UP	1.61E-04	8.25E-03	2.15
HSIAO_LIVER_SPECIFIC_GENES	1.29E-04	7.67E-03	2.13
ELVIDGE_HIF1A_AND_HIF2A_TARGETS_DN	1.35E-04	7.67E-03	2.12
DOANE_BREAST_CANCER_CLASSES_UP	1.44E-04	7.67E-03	2.11
QI_HYPOXIA	1.28E-04	7.67E-03	2.11
DEMAGALHAES_AGING_UP	3.00E-04	1.11E-02	2.09
SMID_BREAST_CANCER_LUMINAL_B_UP	1.29E-04	7.67E-03	2.09
NAKAYAMA_SOFT_TISSUE_TUMORS_PCA2_DN	1.49E-04	7.71E-03	2.08
MARTIN_INTERACT_WITH_HDAC	1.49E-04	7.71E-03	2.08
FARDIN_HYPOXIA_11	3.10E-04	1.11E-02	2.08
ROSS_AML_OF_FAB_M7_TYPE	1.42E-04	7.67E-03	2.06
FARMER_BREAST_CANCER_APOCRINE_VS_LUMINAL	1.16E-04	7.67E-03	2.06
KEGG_ABC_TRANSPORTERS	4.58E-04	1.28E-02	2.04
ONDER_CDH1_TARGETS_2_UP	1.20E-04	7.67E-03	2.04
ELVIDGE_HYPOXIA_UP	1.25E-04	7.67E-03	2.03
HOOI_ST7_TARGETS_DN	1.35E-04	7.67E-03	2.02
SEMENZA_HIF1_TARGETS	3.08E-04	1.11E-02	2.02
ELVIDGE_HYPOXIA_BY_DMOG_UP	1.29E-04	7.67E-03	2.02
FARMER_BREAST_CANCER_APOCRINE_VS_BASAL	1.15E-04	7.67E-03	2.02
ZHANG_TLX_TARGETS_DN	1.37E-04	7.67E-03	2.01
REACTOME_COLLAGEN_FORMATION	2.95E-04	1.11E-02	2.01
BOQUEST_STEM_CELL_UP	1.23E-04	7.67E-03	2.00
FRASOR_TAMOXIFEN_RESPONSE_UP	2.95E-04	1.11E-02	2.00
VALK_AML_CLUSTER_1	4.87E-04	1.31E-02	1.99
BURTON_ADIPOGENESIS_4	2.95E-04	1.11E-02	1.98
TIEN_INTESTINE_PROBIOTICS_6HR_UP	1.31E-03	2.37E-02	-1.98
REACTOME_TRANSLATION	4.78E-04	1.31E-02	-1.99
COLLER_MYC_TARGETS_UP	2.71E-04	1.09E-02	-2.00
REACTOME_SRP_DEPENDENT_COTRANSLATIONAL_PROTEIN_TARGETING_TO_MEMBRANE	4.31E-04	1.23E-02	-2.01
KEGG_SELENOAMINO_ACID_METABOLISM	2.68E-04	1.09E-02	-2.01
XU_HGF_TARGETS_INDUCED_BY_AKT1_6HR	7.59E-04	1.70E-02	-2.01

HECKER_IFNB1_TARGETS	6.69E-04	1.59E-02	-2.03
GAURNIER_PSMD4_TARGETS	5.64E-04	1.43E-02	-2.06
CHNG_MULTIPLE_MYELOMA_HYPERPLOID_UP	6.34E-04	1.54E-02	-2.09
SANA_RESPONSE_TO_IFNG_UP	3.28E-04	1.15E-02	-2.10
REACTOME_INFLUENZA_LIFE_CYCLE	4.82E-04	1.31E-02	-2.11
STAMBOLSKY_TARGETS_OF_MUTATED_TP53_DN	6.10E-04	1.49E-02	-2.13
HALLMARK_INTERFERON_ALPHA_RESPONSE	3.75E-04	1.20E-02	-2.13
ZHANG_INTERFERON_RESPONSE	2.60E-04	1.09E-02	-2.20
REACTOME_ACTIVATION_OF_THE_MRNA_UPON_BINDING_OF_THE_CAP_BINDING_COMPLEX_AND_EIFS_AND_SUBSEQUENT_BINDING_TO_43S	3.17E-04	1.13E-02	-2.28
BOLTON_ANDROGEN_DOWN	3.05E-04	1.11E-02	-2.33
BILANGES_SERUM_AND_RAPAMYCIN_SENSITIVE_GENES	3.40E-04	1.17E-02	-2.33
REACTOME_INTERFERON_ALPHA_BETA_SIGNALING	3.05E-04	1.11E-02	-2.34
BOWIE_RESPONSE_TO_TAMOXIFEN	2.52E-04	1.09E-02	-2.34
REACTOME_NONSENSE_MEDIATED_DECAY_ENHANCED_BY_THE_EXON_JUNCTION_COMPLEX	4.18E-04	1.22E-02	-2.38
REACTOME_FORMATION_OF_THE_TERNARY_COMPLEX_AND_SUBSEQUENTLY_THE_43S_COMPLEX	3.01E-04	1.11E-02	-2.39
REACTOME_INFLUENZA_VIRAL_RNA_TRANSCRIPTION_AND_REPLICATION	4.06E-04	1.20E-02	-2.39
RASHI_NFKB1_TARGETS	2.58E-04	1.09E-02	-2.40
MOSERLE_IFNA_RESPONSE	2.77E-04	1.10E-02	-2.41
FARMER_BREAST_CANCER_CLUSTER_1	2.56E-04	1.09E-02	-2.42
KEGG_RIBOSOME	3.78E-04	1.20E-02	-2.49
EINAV_INTERFERON_SIGNATURE_IN_CANCER	2.68E-04	1.09E-02	-2.52
REACTOME_3_UTR_MEDIATED_TRANSLATIONAL_REGULATION	3.96E-04	1.20E-02	-2.52
BENNETT_SYSTEMIC_LUPUS_ERYTHEMATOSUS	2.68E-04	1.09E-02	-2.55
REACTOME_PEPTIDE_CHAIN_ELONGATION	3.79E-04	1.20E-02	-2.61

Table S3: Top 50 significantly enriched pathways associated with DNA methylation dynamics in HPr1-AR cells exposed to DHT for 96 hours.

pathway	pval	padj	NES
MIKKELSEN_MEF_HCP_WITH_H3K27ME3	1.00E-05	5.70E-04	2.93
MIKKELSEN_MCV6_HCP_WITH_H3K27ME3	1.00E-05	5.70E-04	2.74
BENPORATH_PRC2_TARGETS	1.00E-05	5.70E-04	2.67
MEISSNER_BRAIN_HCP_WITH_H3K27ME3	1.00E-05	5.70E-04	2.66
BENPORATH_ES_WITH_H3K27ME3	1.00E-05	5.70E-04	2.54
MEISSNER_NPC_HCP_WIH_H3K4ME2_AND_H3K27ME3	1.00E-05	5.70E-04	2.51
KONDO_PROSTATE_CANCER_WITH_H3K27ME3	1.10E-05	6.30E-04	2.51
MARTENS_TRETINOIN_RESPONSE_UP	1.00E-05	5.70E-04	2.5
MIKKELSEN_NPC_HPC_WITH_H3K27ME3	1.00E-05	5.70E-04	2.49
RICKMAN_HEAD_AND_NECK_CANCER_A	1.00E-05	5.70E-04	2.49
BENPORATH_SUZ12_TARGETS	1.00E-05	5.70E-04	2.48
NABA_MATRISOME	1.00E-05	5.70E-04	2.45
ZHANG_LUMINAL	1.00E-05	5.70E-04	2.45
KEGG_NEUROACTIVE_LIGAND_RECEPTOR_INTERACTION	1.00E-05	5.70E-04	2.44
NABA_ECM_AFFILIATED	1.00E-05	5.70E-04	2.43
MIKKELSEN_IPS_WITH_HCP_H3K27ME3	1.00E-05	5.70E-04	2.43
NABA_CORE_MATRISOME	1.00E-05	5.70E-04	2.41
ANASTASSIOU_MULTICANCER_INVASIVENESS_SIGNATURE	1.00E-05	5.70E-04	2.36
SABATES_COLORECTAL_ADENOMA_DN	1.00E-05	5.70E-04	2.35
SERVITJA_ISLET_HNF1A_TARGETS_DN	1.00E-05	5.70E-04	2.34
RICKMAN_HEAD_AND_NECK_CANCER_E	2.10E-05	9.60E-04	2.34
HALLMARK_KRAS_SIGNALING_DN	1.00E-05	1.11E-02	2.34
NABA_ECM_GLYCOPROTEINS	1.00E-05	5.70E-04	2.34
MIKKELSEN_ES_LCP_WITH_H3K4ME3	1.00E-05	5.70E-04	2.33
MIKKELSEN_MCV6_LCP_WITH_H3K4ME3	1.00E-05	5.70E-04	2.33
SENGUPTA_NASOPHARYNGEAL_CARCINOMA_WITH_LMP1_DN	1.00E-05	5.70E-04	2.32
MIKKELSEN_IPS_LCP_WITH_H3K4ME3	1.00E-05	5.70E-04	2.32
NABA_MATRISOM_ASSOCIATED	1.00E-05	5.70E-04	2.3
SERVITJA_ISLET_HNF1A_TARGETS_UP	1.00E-05	5.70E-04	2.29
KATSANOU_ELAVL1_TARGETS_UP	1.00E-05	5.70E-04	2.27
LIANG_SILENCED_BY_METHYLATION_2	2.10E-05	9.60E-04	2.26
SMID_BREAST_CANCER_NORMAL_LIKE_UP	1.00E-05	5.70E-04	2.25
KEGG_COMPLEMENT_AND_COAGULATION_CASCADES	4.40E-05	1.80E-03	2.25
SHUETZ_BREAST_CANCER_DUCTAL_INVASIVE_UP	1.00E-05	5.70E-04	2.24
REACTOME_INTERACTION_BETWEEN_L1_AND_ANKYRINS	2.30E-05	1.00E-03	2.24
MIKKELSEN_MEF_LCP_WITH_H3K4ME3	1.00E-05	5.70E-04	2.24

SATO_SILENCED_EPIGENETICALLY_IN_PANCREATIC_CANCER	2.10E-05	9.60E-04	2.24
REACTOME_CLASS_A1_RHODOPSIN_LIKE_RECEPTORS	1.00E-05	5.70E-04	2.23
ZHANG_ANTIVIRAL_RESPONSE_TO_RIBAVIRIN_UP	6.60E-05	2.40E-03	2.23
TAKEDA_TARGETS_OF_NUP98_HOXA9_FUSION_10D_UP	1.00E-05	5.70E-04	2.22
NAVA_ECM_REGULATORS	1.00E-05	5.70E-04	2.21
MIKKELSEN_MEF_ICP_WITH_H3K27ME3	2.00E-05	9.60E-04	2.2
MEISSNER_NPC_HCP_WITH_H3K27ME3	3.10E-05	1.40E-03	2.2
REACTOME_G_ALPHA_S_SIGNALLING_EVENTS	2.00E-05	9.60E-04	2.18
MUELLER_METHYLATED_IN_GLIOBLASTOMA	6.40E-05	2.30E-03	2.18
WANG_MLL_TARGETS	1.00E-05	5.70E-04	2.18
REACTOME_POTASSIUM_CHANNELS	5.20E-05	2.00E-03	2.17
MIKKELSEN_ES_ICP_WITH_H3K4ME3	1.00E-05	5.70E-04	2.15
REN_ALVEOLAR_RHABODMYOSARCOMA_UP	4.10E-05	1.70E-03	2.14
REACTOME_GPCR_LIGAND_BINDING	1.00E-05	5.70E-04	2.14

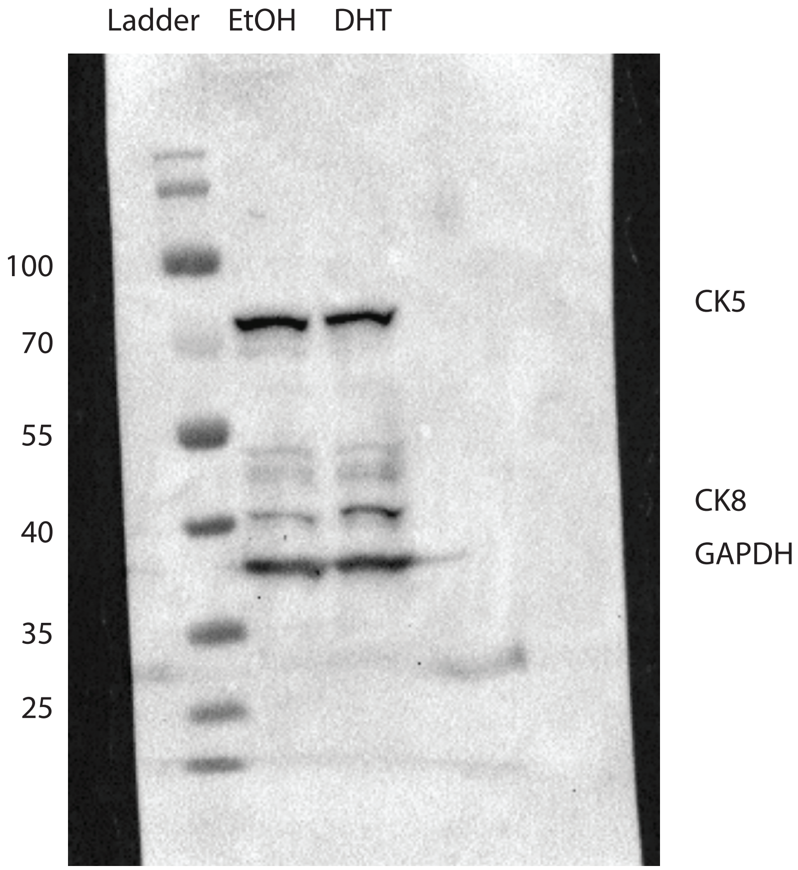
Ladder
EtOH
DHT
100
70
55
40
35
25
CK5
CK8
GAPDH
